# A novel study for producing complexed and encapsulated nutrients at nanometric scale to enhance plant growth

**DOI:** 10.1038/s41598-023-37607-x

**Published:** 2023-07-09

**Authors:** Marium Khaliq, Muhammad Asif Hanif, Ijaz Ahmad Bhatti, Zahid Mushtaq

**Affiliations:** 1 Nano and Biomaterials Lab, Department of Chemistry, University of Agriculture, Faisalabad, 38040 Pakistan; 2 Department of Biochemistry, University of Agriculture, Faisalabad, 38040 Pakistan

**Keywords:** Nanoscale materials, Chemical safety, Green chemistry

## Abstract

Complexation of micronutrients with complexing agents reduce undesirable reactions of fertilizers in soil water system. In the form of complex structure nutrients remain available to plants in the useable form. Nanoform fertilizer enhances the surface area of particles and less amount of fertilizer contact with large area of plant roots which reduce fertilizer cost. Controlling release of fertilizer using polymeric material like sodium alginate makes agriculture practices more efficient and cost effective. Several fertilizers and nutrients are used at a large scale to improve crop yields globally and almost more than half goes to waste. Therefore, there is a dire need to improve plant-available nutrients in soil, using feasible, environmentally friendly technologies. In the present research, complexed micronutrients were successfully encapsulated using a novel technique at nanometric scale. The nutrients were complexed with proline and encapsulated using sodium alginate (polymer). Sweet basil was subjected to seven treatments over three months in a moderately controlled environment (25 °C of temperature and 57% of humidity) to study the effects of synthesized complexed micronutrient nano fertilizers. The structural modifications of the complexed micronutrient nanoforms of fertilizers were examined, through X-ray powder diffraction (XRD) and scanning electron microscopy (SEM). The size of manufactured fertilizers was between 1 and 200 nm. Fourier transform infrared (FTIR) spectroscopy stretching vibration peaks at 1600.9 cm^−1^ (C=O), 3336 cm^−1^ (N–H) and at 1090.2 cm^−1^ (N–H in a twisting and rocking) corresponds to the pyrrolidine ring. Gas chromatography–mass spectrometry was used to analyze the chemical makeup of the essential oil of the basil plants. Essential oil yield of basil plants increased from 0.0035 to 0.1226% after treatments. The findings of the present research show that complexation and encapsulation improve crop quality, essential oil yield, and antioxidant potential of basil.

## Introduction

Due to leaching, heavy cropping, liming of acidic soil and topsoil erosion, micronutrient deficits in crops have increased significantly during the past few years^1^. Low crop quality and yield, widespread different pest and diseases infestation, imperfect morphological structure of plant (like small size fewer small xylem vessels), low phytosiderophores activation, and decreased use efficiency of fertilizers are some of the negative effects caused by the deficiency of micronutrients in plants^2^. Even though crop plants need micronutrients in lower concentrations, they are essential to the growth as well yield of many crops^3^. The above-mentioned problems may be resolved by using micronutrient fertilizers in chelated forms^3^. Root nodes of plants possess slightly negative charge and metal ions of micronutrients are electropositive in nature, so they bind with root node sites and do not flow into the plant tissues. As these nutrients combine with a complexing agent, they become neutral or slightly negative so pass through the plant tissues easily. Proline is an effective bidentate ligand^4^. It protects the plant from a variety of challenges and aids in their quicker recovery from stress. Proline increases plant growth as well as other physiological characteristics when it is administered exogenously to stressed plants^5^. Nutrients that are supplied to plants in the form of fertilizers are crucial for appropriate plant growth and their metabolism but inappropriate supply of fertilizers to crops brings about 40–70% drainage of fertilizer cause contamination of heavy metals in fresh and ground water reservoirs. Nano-fertilizers provide nutrients precisely to the plant’s requirement, and thus reduces the environmental loss of nutrients^6^. The most important and powerful technique is the development of nanotechnology for the controlled release of fertilizers and pesticides in the agriculture fields. Development of nanocarriers, nano fertilizers, and nanosensors has improved fertilizer efficiency with minimum wastage^7^. Nanotechnology has been found to be quite successful for the synthesis of controlled release formulations of agrochemicals^8^. The benefits of controlled release technology include decreased need for active agents, and longer persistence of active agents in the water-soil system which makes agricultural methods more cost-effective. Additionally, this protects the groundwater from the hazardous pesticides, insecticides, and other chemicals that have been used^9^. The use of nanocarriers, which behave as vehicles of the necessary micronutrients and deliver them with required quantity as well as time duration, is one of the viable techniques to tackle the micronutrients deficiency^10^. The usage of naturally occurring polymers has significantly increased in recent years because of non-toxicity, abundance in nature^11^, easy availability^12^, low cost^13^, ecofriendly nature^14^, biodegradability^15^, and ease of functionalization. The studies reporting the usage of biopolymers such sodium alginate, chitosan, starch and polysaccharide are well-documented in the literature^16^. Aromatic plants have utilization in several industries^17,18^ and plants like basil are quick responsive towards fertilizer applications.

In the present study, experiments were carried out to study the application of nutrient fertilizers on basil yield in which proline was used as a complexing agent and sodium alginate as an immobilization material. The novelty of present work is the production of immobilized and complexed fertilizers that exhibited cost effectiveness and enhancement of crop production by increasing soil fertility with balanced nutrients availability. These studies will be helpful for improving fertilizer recommendations and for achieving sustainable productions in basil.

## Materials and methods

### Cultivation of basil plants

For the *Ocimum* *basilicum* plant proper growth, coconut coir was employed as the growth medium. Table 1 displays the composition of coconut coir, whereas Table 2 lists its physical characteristics. The pots were filled with thoroughly mixed soil, sand, and coconut coir in ratios of 3:3:1. Sand was used with the intention of softening the soil and promoting healthy root growth. The seeds of *Ocimum* *basilicum* were bought from the Faisalabad market. To grow basil seeds, a seedling tray was filled with mixed soil and two seeds per cell were sown at 1 cm depth. The seedling tray was covered with a clear plastic bag and soil was kept moist during the growth of seeds. *Ocimum* *basilicum* seedlings that were in good health were transplanted into pots of 20-in. at the age of four weeks (one seedling per pot) to allow for optimal plant growth and to enhance the amount of total moisture available. The humidity and temperature were maintained uniformly for all of the pots. The experiments were conducted at University of Agriculture, Faisalabad, Pakistan, using a randomized complete block design in a Greenhouse having light intensity of 500 μmol/m^2^/s at 25 °C of temperature and 57% of humidity^19^. All the experiments were run in replicates (four replicates of each treatment). There was a total of seven treatments, each having four plants.

**Table 1:** Coconut/coir fiber chemical composition.

Sr. no.	Chemicals	Percentage (%)
1	Lignin	45.84
2	Ash	02.22
3	Pectin’s and related compound	03.00
4	Hemi-cellulose	00.25
5	Water soluble	05.25
6	Cellulose	43.44

**Table 2:** Coconut/coir fiber physical properties.

Sr. no	Properties	
1	Length in inches	6–8
2	Tenacity (g/Tex)	10.0
3	Density (g/cc)	1.40
4	Diameter in mm	0.1–0.5
5	Breaking elongation %	30
6	Moisture at 65% RH	10.50
7	Swelling in water (diameter) in %	5
8	Rigidity of modulus in dyne/cm^2^	1.8924

### Preparation of fertilizers

The quantities listed in Table 3 were used to make the solutions of macronutrient (Sigma Aldrich) nutrition separately^20^. Blank solution (T1), control solution for non-immobilized micronutrients fertilizer (T2), control solution for immobilised micronutrients fertiliser (T3), and two types of complexed micronutrients nano fertiliser (i) non-immobilized proline micronutrients nano fertilizer NI/Pro-MNF (T4, T5) and (ii) immobilised proline micronutrients nano-fertilizer I/Pro-MNF (T6, T7) were prepared in order to test the treatment of targeted fertilizers with them.

**Table 3:** The macronutrients concentrations used in the current study^20^.

Sr. no	Macronutrients	g/L
1	KH_2_PO_4_	27.21
2	MgSO_4_	98.59
3	Ca(NO_3_)_2_	236.16
4	KNO_3_	101.11

### Preparation of stock

The following Table 4 lists the levels of micronutrients (Sigma Aldrich) used in the current investigation^20^. The micronutrients were mixed with two different levels including 5 g and 7.5 g of proline (Sigma Aldrich) complexing agent^20,21^. This solution was thoroughly mixed and then dehydrated in an electric oven at 150˚C. The fertiliser was allowed to slowly cool down to lab temperature after drying at temerature of 150˚C. After cooling, final hard mass material was grinded into a fine powder using a ball mill to nano-metric range (mesh size 1–1000 nm)^6^. Non-immobilised micronutrients nano fertiliser with 5 g proline, non-immobilised micronutrients nano fertiliser with 7.5 g proline, immobilised micronutrients nano fertiliser with 5 g proline, and immobilised micronutrients nano fertiliser with 7.5 g proline were designated as T4, T5, T6 and T7 respectively (Table 5).

**Table 4:** The micronutrients concentrations used in the current study.

Sr. no	Chemicals	Amount (g/L)
1	H_3_BO_3_	2.86
2	ZnSO_4_⋅7H_2_O	0.22
3	CuSO_4_⋅5H_2_O	0.08
4	NH_4_)_6_MoO_24_⋅2H_2_O	0.02
5	MnCl_2_⋅H_2_O	1.82

**Table 5:** The fertlizer treatments used in the present study.

Treatment	Chemicals	Proline amount (g)
T1	Blank (no chemicals addition)	0
T2	Control (micronutrients only)	0
T3	Control immobilized (micronutrients only)	0
T4	Micronutrients + proline → non-immobilized	5.0
T5		7.5
T6	Micronutrients + proline → immobilized	5.0
T7		7.5

### Perparation of non-immobilzed complexed micronutrients nano fertilizer

In order to prepare T2, T4 and T5 from mixture, 1 g of each level was diluted with 1 l of distilled water. The experiment lasted 3 months, and every week, 100 ml of prepared fertiliser was applied to the each plant.

### Preparation of immobilized complexed micronutrients nano fertilizer

**Figure 1:**
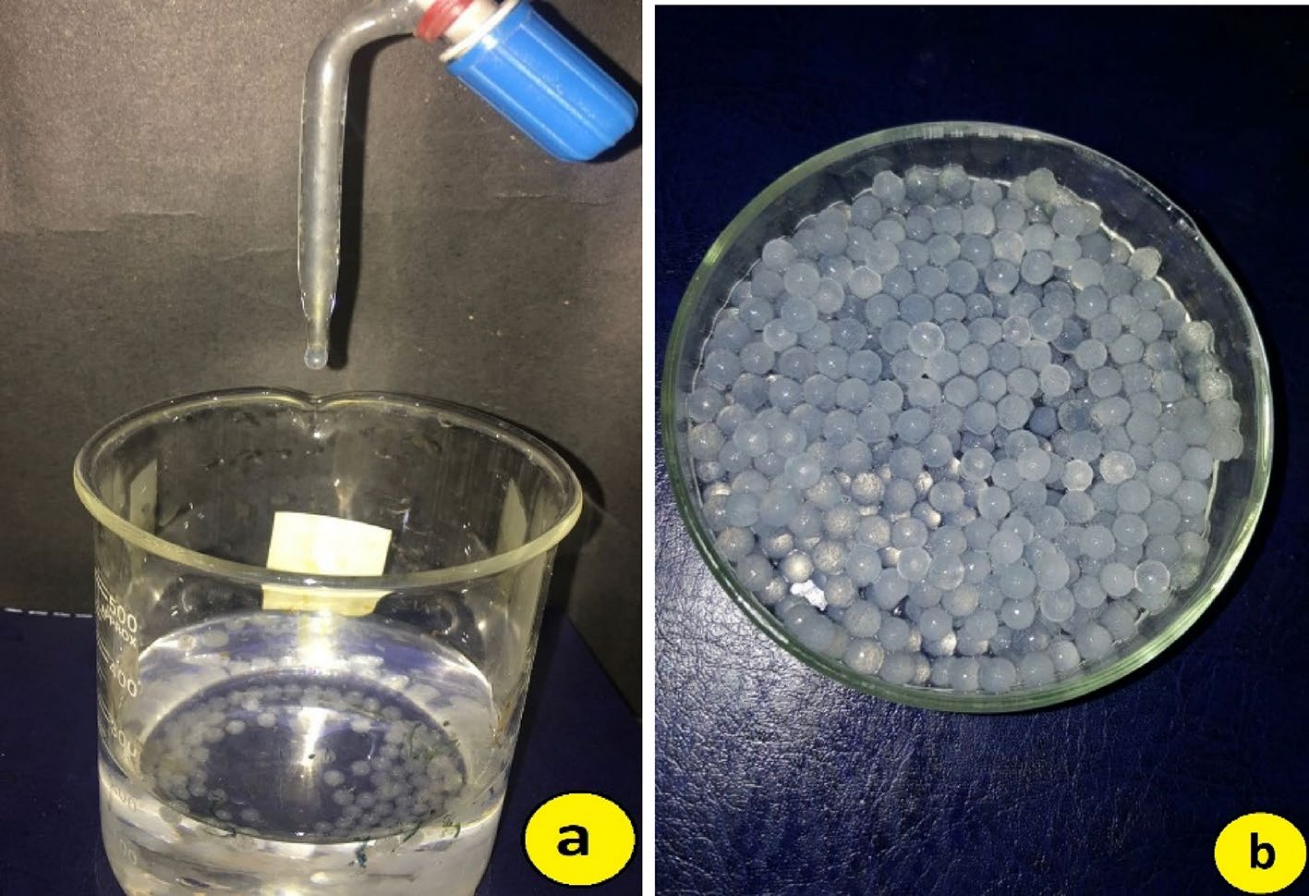
(**a**) Set up to prepare nano-carriers, (**b**) prepared nano-carriers.

For the preparation of immobilized or encapsulated form of nano fertilizers for treatments T3, T6, and T7, sodium alginate (Sigma Aldrich) micro-emulsion was prepared by adding 1 g of sodium alginate into 30 ml distilled water containing 3–4 drops of paraffin oil. This mixture was vigorously stirred for 40 min and then added 1.2 g of nano fertilizer from stock. This thick paste was added in a burette and droplets were allowed to fall in 1 M calcium chloride (Sigma Aldrich) solution^22^ which turned into 300 solid beads (Fig. 1) that were applied once to each plant as a single dose for three months.

### Characterization of synthesized fertilizers

The prepared proline-complexed micronutrient nano-fertilizers were characterized by several techniques including.

#### (a) X-ray diffraction (XRD)

The phases as well as crystallinity of prepared samples T2, T3, T4, and T6 were examined using the X-ray diffraction (XRD). Samples were dried and finely grounded through ball mills. The Scherrer Equation, L = Kλ/β. cosθ, was used for calculating the size of nano crystallite (L). Brucker D8 Advance diffractometer was employed for this purpose^23^.

#### (b) Fourier transforms infrared (FT-IR) spectroscopy

The Spectrum GX FT-IR spectrometer (Perkin Elmer, USA) was used to conduct FT-IR (Fourier transforms infrared) analysis of synthesized nano-fertilizers samples T2, T3, T4, and T6. The samples were performed by accumulating a total of 32 scans at a wavenumber of 4000–400 cm^−1^ with a resolution of 4 cm^−1^ were gathered for this purpose. FT-IR analysis was conducted using potassium bromide as the matrix^22,24,25^.

#### (c) Scanning electron microscopy (SEM)

SEM (scanning electron microscopy) (Nova NanoSEM) was used to assess the shape, surface morphology, behavior, and analysis of synthesized nano-fertilizers T2, T3, T4, and T6.

### Plant measurements

Following the application of fertilizers, measurements were taken of all plants^26^. There was a total of seven treatments, each having four plants.

#### Proximate analysis

For each treatment, the plant's weight, height, moisture contents and ash contents were estimated. Harvested plants were dried at 60 °C in an electric oven, until the sample weight remained consistent^26^. Then, it was ground to a fine powder and properly stored for further use^27^.

#### Extraction of essential oil (EO)

Clevenger type hydro distillation equipment was used to assess the essential oil (EO) yield of basil plants treated with synthetic non-immobilized and immobilized complexed micronutrients nano-fertilizer^28–30^. Weighed basil plant material was soaked in water in a round bottom flask for EO extraction^31,32^. The EO yield was calculated with the help of the following formula and Tukey HSD test was applied on the data

$$Percentage\,Yield=\frac{weight\,of\,oil\,extracted}{weight\,of\,sample\,used\,for\,extraction}\times\,100$$

#### Evaluation of biological properties of treated plants

Biological activities such as antioxidants^33^ and insecticidal activities^34^ of all plants, after applying seven different treatments were evaluated according to standard methos given in literature. These activities were evaluated by preparing the extract of basil plants with methanol.

### GC–MS analysis

Basil EO (0.1 µl) was injected into a GC–MS (QP-2000 instrument equipped with an HP 597A mass selective detector and capillary column of Ulbon HR1) under the following conditions: helium was used as the carrier gas, flowing at a rate of 1.5 ml/min with a temperature range of 70 to 225 °C (100 °C/min); the injector and detector temperatures were 250 and 280 °C, respectively. The mass spectrometry conditions were as follows: mass range of 0–400 Da, ionization voltage of 70 eV, and emission current of 40 mA^35^. Unknown chemicals were identified by comparing the observed spectra with mass spectrum libraries.

### Experimental research and permission statement

It is submitted that the experimental research on plants, including the collection of plant material, complied with relevant institutional, national, and international guidelines and legislation. The plant collection procedures/permission and all other protocols were approved by Scrutiny committee of Department of Chemistry, University of Agriculture, Faisalabad, Pakistan.

## Results and discussions

### Characterization of treated plants

#### Proximate analysis of treated basil plant

All plants' height, biomass, moisture, ash, and percentage oil yield were assessed, and the results obtained are shown in Table 6. All treatments (T2–T7) have shown higher biomass contents and better essential oil yield (%) than blank. In all treatments, T7 have shown highest percentage of essential oil. According to Table 7 the p-value corresponding to the F-statistic of one-way ANOVA is lower than 0.05, suggesting that the one or more treatments are significantly different. To identify which of the pairs of treatments are significantly different from each other Tukey HSD test was applied on the data. The p-value corresponding to the Tukey HSD test is lower than 0.01 for all treatments suggesting that all pairs of treatments are significantly different. Maximum and minimum moisture concentrations were recorded in T1 and T6, respectively. T6 had the highest ash contents and T2 had the lowest^27^.

**Table 6:** Proximate analysis of treated plants.

Treatments	Plant height (cm)	Biomass (g)	Moisture contents (g) from 25 g plant material	Ash contents (g) from 1 g powdered plant material	Oil yield (%)
T1	13.0 ± 0.10	193 ± 7	18.59 ± 0.13	0.030 ± 0.002	0.0035 ± 0.003
T2	15.0 ± 0.09	208 ± 8	18.15 ± 0.11	0.005 ± 0.001	0.0190 ± 0.002
T3	17.0 ± 0.08	252 ± 9	17.87 ± 0.16	0.060 ± 0.007	0.0098 ± 0.005
T4	18.0 ± 0.11	310 ± 8	18.55 ± 0.19	0.100 ± 0.003	0.0557 ± 0.008
T5	18.5 ± 0.09	290 ± 7	17.70 ± 0.17	0.110 ± 0.005	0.0620 ± 0.009
T6	16.5 ± 0.13	350 ± 9	16.14 ± 0.11	0.130 ± 0.004	0.0467 ± 0.006
T7	16.0 ± 0.08	270 ± 6	17.06 ± 0.14	0.120 ± 0.008	0.1226 ± 0.007

**Table 7:** Descriptive statistics for essential yield obtained after various treatments.

(A) One-way ANOVA								
Treatment →	T1	T2	T3	T4	T5	T6	T7	Pooled total
Observations N	3	3	3	3	3	3	3	21
Sum ∑xi	0.0105	0.0570	0.0293	0.1668	0.1860	0.1400	0.3678	0.9574
Mean x	0.0035	0.0190	0.0098	0.0556	0.0620	0.0467	0.1226	0.0456
Sum of squares ∑xi2	0.0000	0.0011	0.0003	0.0093	0.0115	0.0065	0.0451	0.0738
Sample variance s2	0.0000	0.0000	0.0000	0.0000	0.0000	0.0000	0.0000	0.0015
Sample std. dev. s	0.0001	0.0001	0.0001	0.0001	0.0001	0.0001	0.0001	0.0389
Std. dev. of mean SEx	0.0001	0.0001	0.0000	0.0001	0.0001	0.0000	0.0001	0.0085

Source	Sum of squares SS	Degrees of freedom ν	Mean square MS	F statistic	p-value			
Treatment	0.0302	6	0.0050	621,544.0981	1.1102e−16			
Error	0.0000	14	0.0000					
Total	0.0302	20						

(B) Tukey HSD results								
Treatments pair	Tukey HSD Q statistic	Tukey HSD p-value	Tukey HSD inference					
T1 vs T2	298.3854	**0.0010053**	**p < 0.01					
T1 vs T3	120.6375	**0.0010053**	**p < 0.01					
T1 vs T4	1002.9598	**0.0010053**	**p < 0.01					
T1 vs T5	1126.1641	**0.0010053**	**p < 0.01					
T1 vs T6	830.9872	**0.0010053**	**p < 0.01					
TI vs T7	2292.7546	**0.0010053**	**p < 0.01					
T2 vs T3	177.7478	**0.0010053**	**p < 0.01					
T2 vs T4	704.5745	**0.0010053**	**p < 0.01					
T2 vs T5	827.7787	**0.0010053**	**p < 0.01					
T2 vs T6	532.6018	**0.0010053**	**p < 0.01					
T2 vs T7	1994.3693	**0.0010053**	**p < 0.01					
T3 vs T4	882.3223	**0.0010053**	**p < 0.01					
T3 vs T5	1005.5266	**0.0010053**	**p < 0.01					
T3 vs T6	710.3497	**0.0010053**	**p < 0.01					
T3 vs T7	2172.1171	**0.0010053**	**p < 0.01					

Bold shows all treatment were significantly different.

#### GCMS analysis of the EO extracted from the biomass of plant

**Figure 2:**
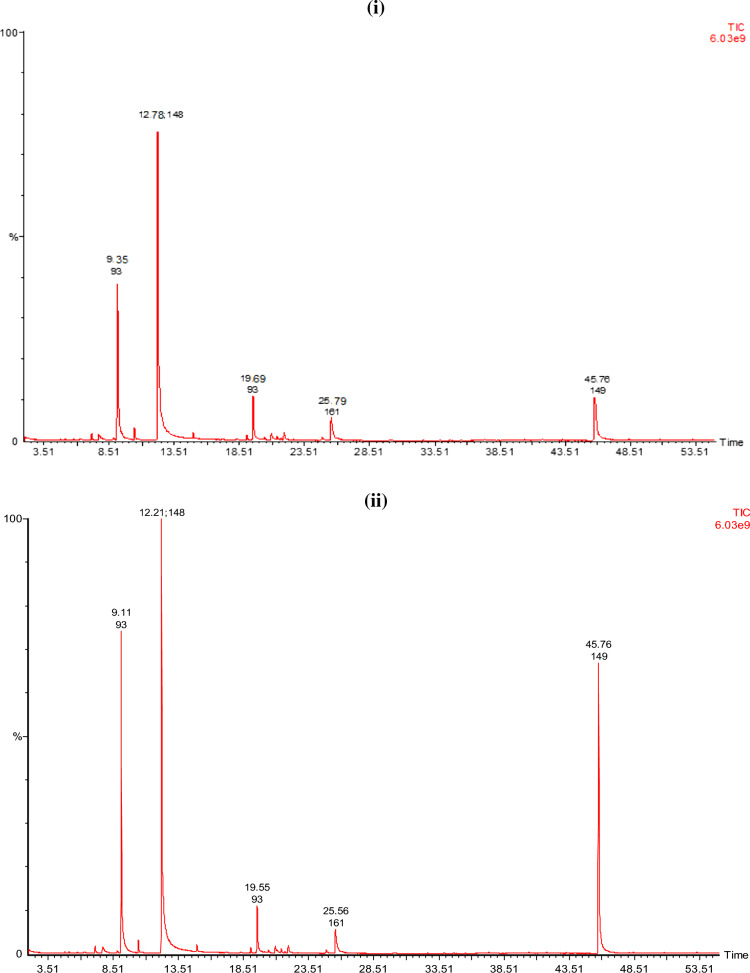

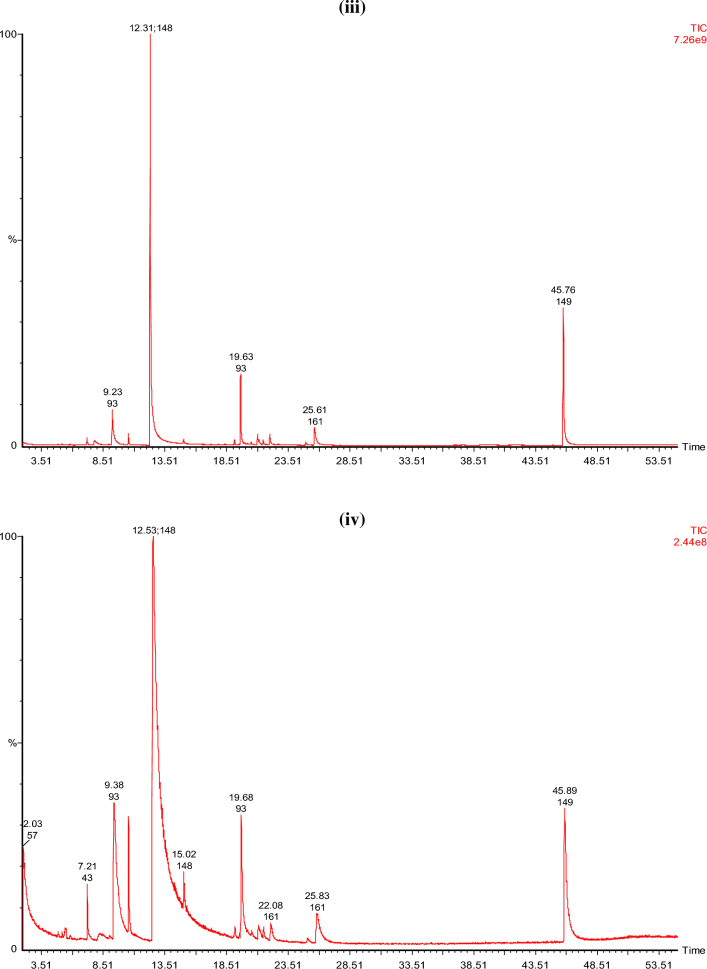

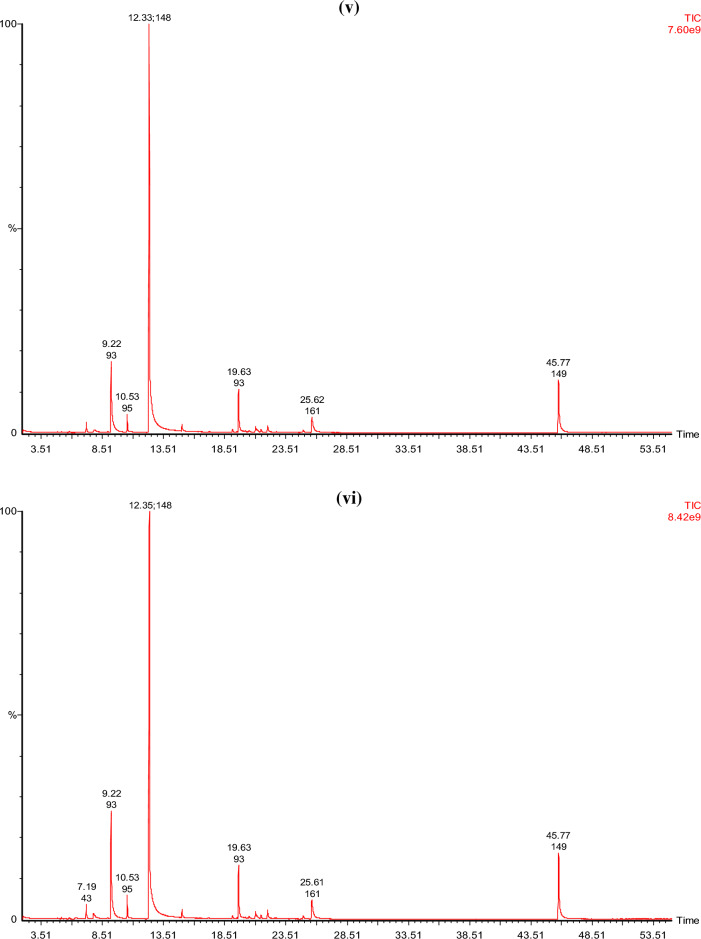

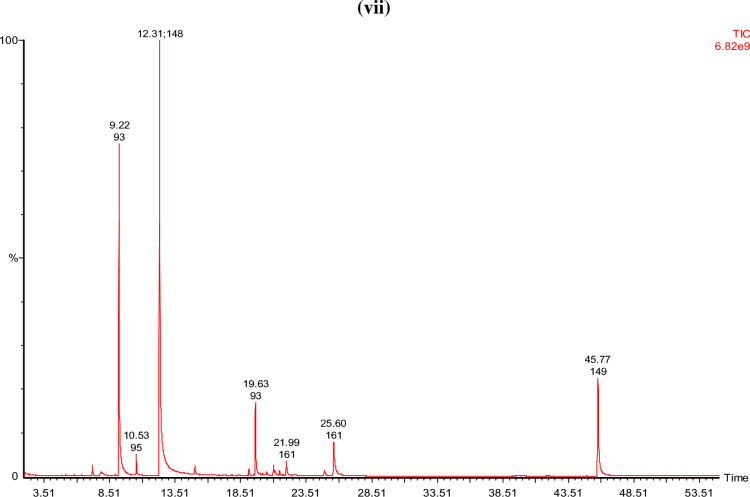
GCMS analysis of basil essential oil **(i)** T1 (blank), **(ii)** T2 (control), **(iii)** T3 (control immobilized), (iv) T4 [proline (5 g)], **(v)** T5 [proline (7.5 g)], **(vi)** T6 [proline immobilized (5 g)], **(vii)** T7 [proline immobilized (7.5 g)].

The results of GC–MS analysis of basil essential oil produced after all treatments are given in Fig. 2. Treatments (T2–T7) found to have a greater number of compounds than blank (T1)^36,37^. In essential oil produced from basil plants of T1, T2, T3, T4, T5, T6, and T7 identified chemical constituents were 16, 24, 24, 18, 22, 22, and 23, respectively. Estragole was found to be the primary component in the EO oils of all basil plants that had been treated. Estragole was found to have a maximum concentration in treatment T5, and a minimum concentration in treatment T1 (blank). Estragole levels also varied with immobilization of fertilizer^38–40^. The commercial cultivars of 'Sweet Basil' have known to contain methyl chavicol (estragole), eugenol, linalool and 8-cineole as their primary essential oil constituents^41^. Depending on the season, location, and fertilizer used on the plant, the ratio of the various EO components changes greatly^42–44^. Estragole, a phenylpropanoid derivative, is commonly present in various plants as well as *Ocimum* *basilicum* (sweet basil)^45^. Which is a naturally occurring substance that may be extracted from fennel, star anise, anise and basil. Flavors as well as fragrances that have estragole are widely used in perfumes, food items, detergents and soaps. According to the Flavor and Extract Manufacturers Association (FEMA), estragole exposure in the USA is estimated to be 70 µg per capita per day^46^. Estragole, on the other hand, significantly affects the overall aroma of the *Ocimum* *basilicum*.

Proline has a number of characteristics that contribute to its capacity to improve plant resistance. (i) Proline, a potent osmolyte, can increase cellular osmotic pressure^47^. (ii) Proline protects against oxidative damage. One of the earliest plant responses to biogenic and abiogenic stresses is generally recognized to oxidative stress (an increase in the concentration of reactive oxygen species (ROS))^48^. A proline molecule's structure enables direct interactions with several types of ROS, which inactivates them and lowers their levels. Additionally, proline can reduce oxidative stress by triggering the antioxidant enzymes catalase, ascorbate peroxidase and superoxide dismutase^49^. (iii) Proline functions as a chelator of metals, forming non-toxic compounds with them. (iv) Proline functions similarly to protein chaperones, heat shock proteins, in that it can stop stress-induced protein denaturation as well as aggregation while also stabilizing cellular structures. Proline prevents proteins from becoming denaturized when it interacts with antioxidant enzymes^5,50^, and other proteins^51,52^. It can also exert indirect protective effects on protein structure by regulating the actions of chaperones themselves^53^. (v) Proline is a proteinogenic in nature mean it can participate in protein synthesis. It causes rigidity as well as stability of a protein's structure in a region of a "fracture" when it is positioned inside protein’s alpha helical and beta-banded segments. This characteristic is supposed to guard enzymes against unspecific proteolytic breakdown. Proline plays a role in the synthesis of proline rich proteins (PRPs), which support the function of the cell walls as barriers against pathogens and unfavorable environmental conditions. (vi) Proline performs signaling tasks by triggering the production of the genes that code for the enzymes which help plants against stressors. For instance, it can activate the genes for antioxidant enzymes (catalase, ascorbate peroxidase, superoxide dismutase, etc.)^54^.

### Biological properties of treated plants

#### Antioxidant activities

According to the findings, higher TPC (total phenolics contents), TFC (total flavonoids contents), DPPH (α-diphenyl-β-picrylhydrazyl), and RPA (reducing power activity) activities were shown by T5, T6, T6 and T4, respectively. Treatment (T2) has shown lowest TPC, TFC, DPPH, and RPA activities after blank. The antioxidant potential of any sample is dependent on substitution, configuration, and total number of hydroxyl (OH) groups; the functional groups arrangement around the nuclear structure; and the total number, structure, and occurrence of antioxidant active components. In previous studies, it was also observed that basil plants have strong antioxidant properties^55^.

#### Insecticidal activities

**Figure 3:**
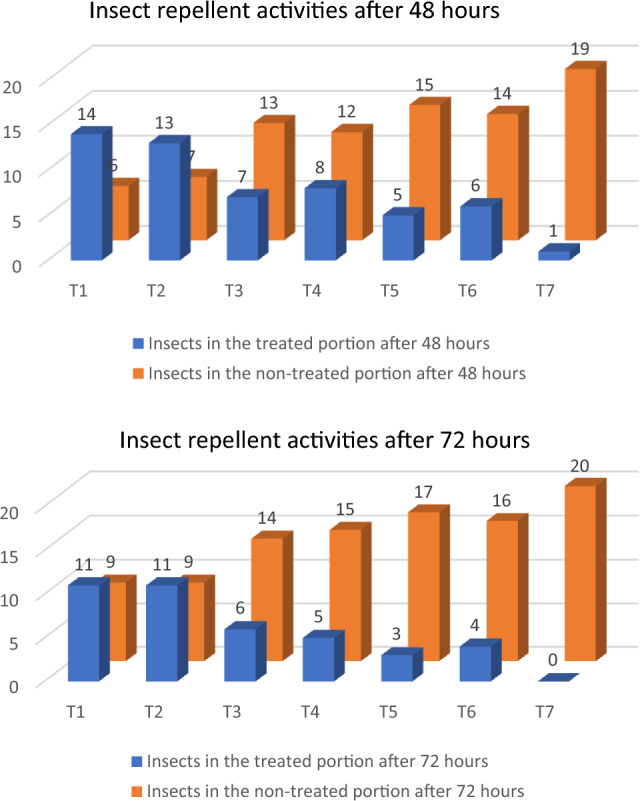
Insect repellent activities of metanolic extract of treatred basil plants after 48 and 72 h.

Khapra beetles were exposed to methanolic extracts of basil plants that had been treated in the current study to test the insecticidal effects. Figure 3 displays the repellency of khapra beetles against the seven basil plant extracts. According to the findings, T1 and T2 had the lowest repellent activity whereas T7 had the maximum repellent activity after 48 h of exposure. After 72 h, similar effects were attained. The insecticidal activities of this extract were shown to be caused by the monoterpene molecules found in the essential oil. Insect-repelling activities of certain EO and their isolated compounds had previously been observed^56^. Main components responsible for insecticidal activities of plant extracts and EO are monoterpenoids^57^. It was observed that the current results are consistent with earlier ones^58^.

### Characterization of fertlizers

#### FTIR analysis

**Figure 4:**
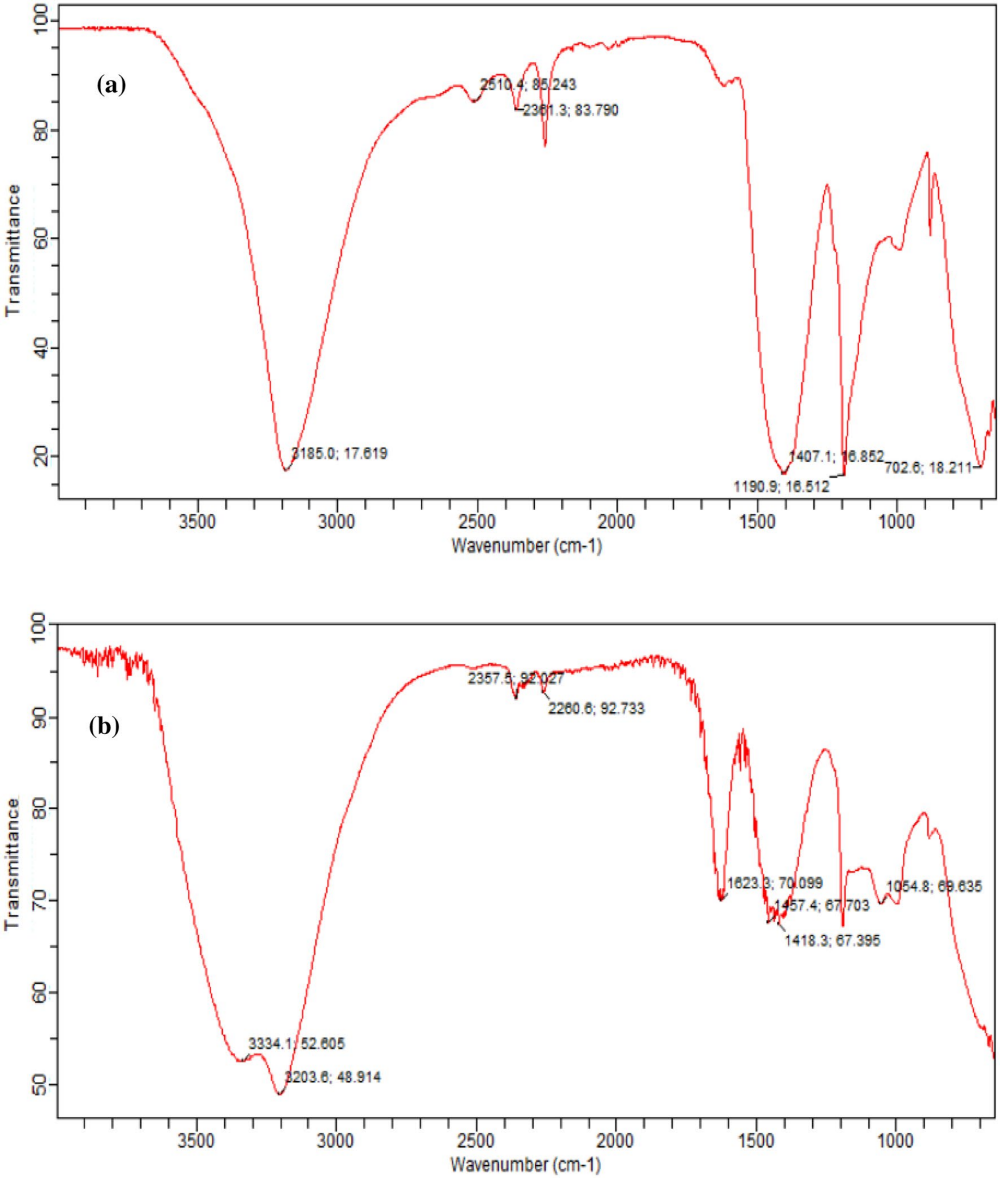
FTIR spectra of (**a**) control (T2) and (**b**) immobilized control nano fertilizer (T3).

Figure 4a, b show the FTIR spectrum of immobilized control nano-fertilizer and non-immobilized control nano-fertilizer, respectively. The peaks of both the a and b spectra of Fig. 4 can be seen to clearly differ from one another, demonstrating the successful modification of the synthesized nano-fertilizers. These fertilizers were synthesized without the complexing agent proline to evaluate the impact of complexed nano-fertilizers (CNF) with these nano-fertilizers. The spectrum displays several peaks, indicating the presence of diverse functional groups in the produced nano-fertilizer. The major goal of performing an FTIR study on synthesized nano-fertilizers was to determine the effect of immobilization. The incorporation of nutrients in immobilized material can be explained by the peak shifting in the FTIR spectra to 3334.1 cm^−1^, 2357.5 cm^−1^, 2260.8 cm^−1^, 1623.3 cm^−1^, 1418.3 cm^−1^, and 1054.8 cm^−1^^59^. sodium alginate's stretching vibration of C=O showed a peak at 1000–1100 cm^−1^^60^. The Na–O bond vibration was associated with the peak that appeared at 1000 cm^−1^.

**Figure 5:**
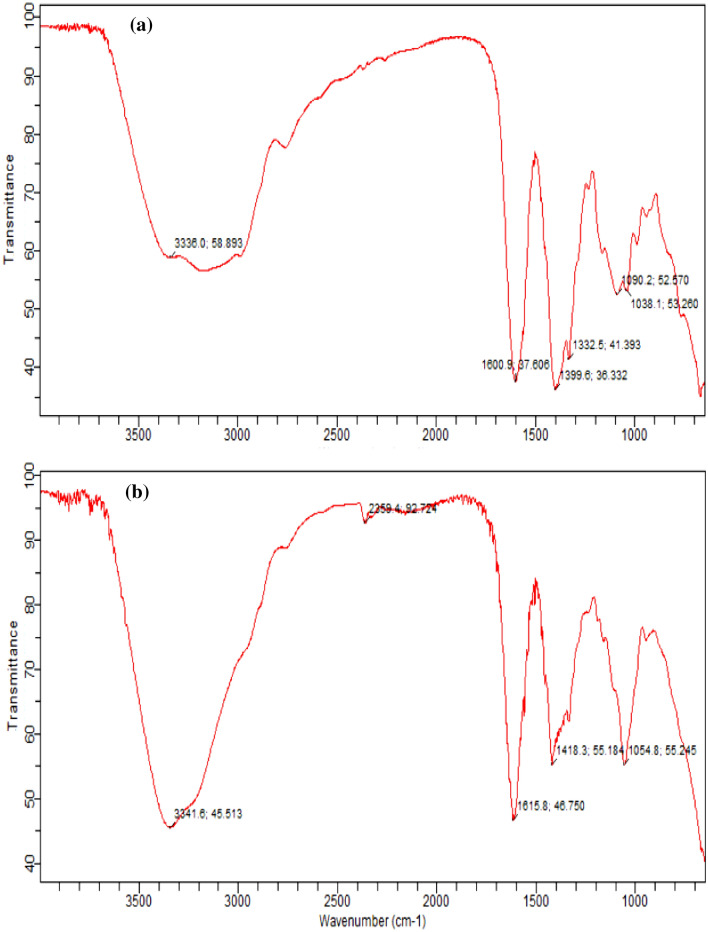
FTIR spectra of (**a**) NI/Pro-MNF (T4) and (**b**) I/Pro-MNF (T6).

FTIR spectrum of non-immobilized complexed micronutrient with proline nano fertilizer (NI/Pro-MNF) and immobilized complexed micronutrient with proline nano fertilizer (I/Pro MNF) is shown in Fig. 5a, b. There is a clear difference between the peaks of both a and b spectra of Fig. 5. Multiple peaks appeared in the spectrum show that the synthesized nano-fertilizer has a variety of function groups. The stretching vibrations of C=O and N–H are responsible for the peaks in the FTIR spectra seen at 1600.9 cm^−1^ and 3336 cm^−1^^61^. However, the peak of NI/Pro-MNF's FTIR spectrum that appeared at 1090.2 cm^−1^ corresponds to the pyrrolidine ring's N–H in a twisting and rocking motion. Other researchers^62^ have also reported similar findings. The wavenumber area between 900 and 1100 cm^−1^ have shown stretching frequencies of metal–oxygen and the range between 1100 and 1150 cm^−1^ have shown stretching frequencies of metal-nitrogen. The peak shifting in the FTIR spectrum of I/Pro-MNF (Fig. 5b) to 3341.6 cm^−1^, 1615.8 cm^−1^, and 1054.8 cm^−1^ was attributed to the incorporation of nutrients into the immobilized substance^59^. Sodium alginate's stretching vibration of C=O showed a peak at close to 1000–1100 cm^−1^^60,63^ and the Na–O bond vibration was associated with the peak that appeared at 1000 cm^−1^^64^.

#### Scanning electron microscopy (SEM) analysis

**Figure 6:**
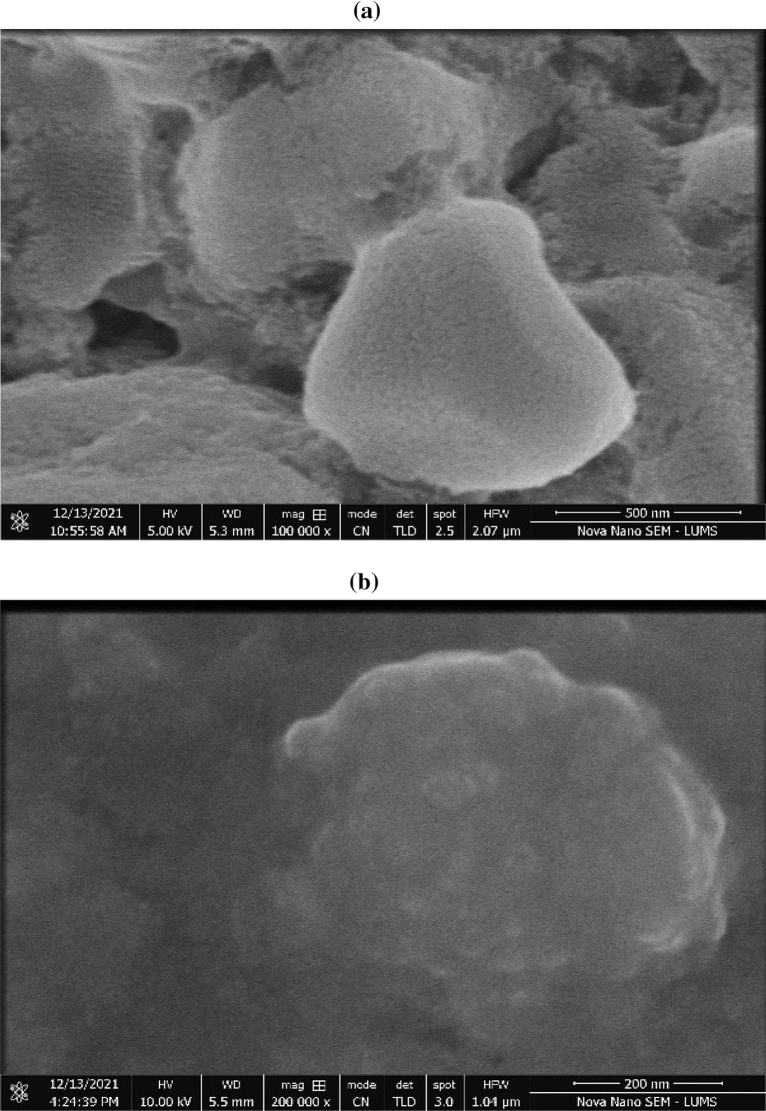

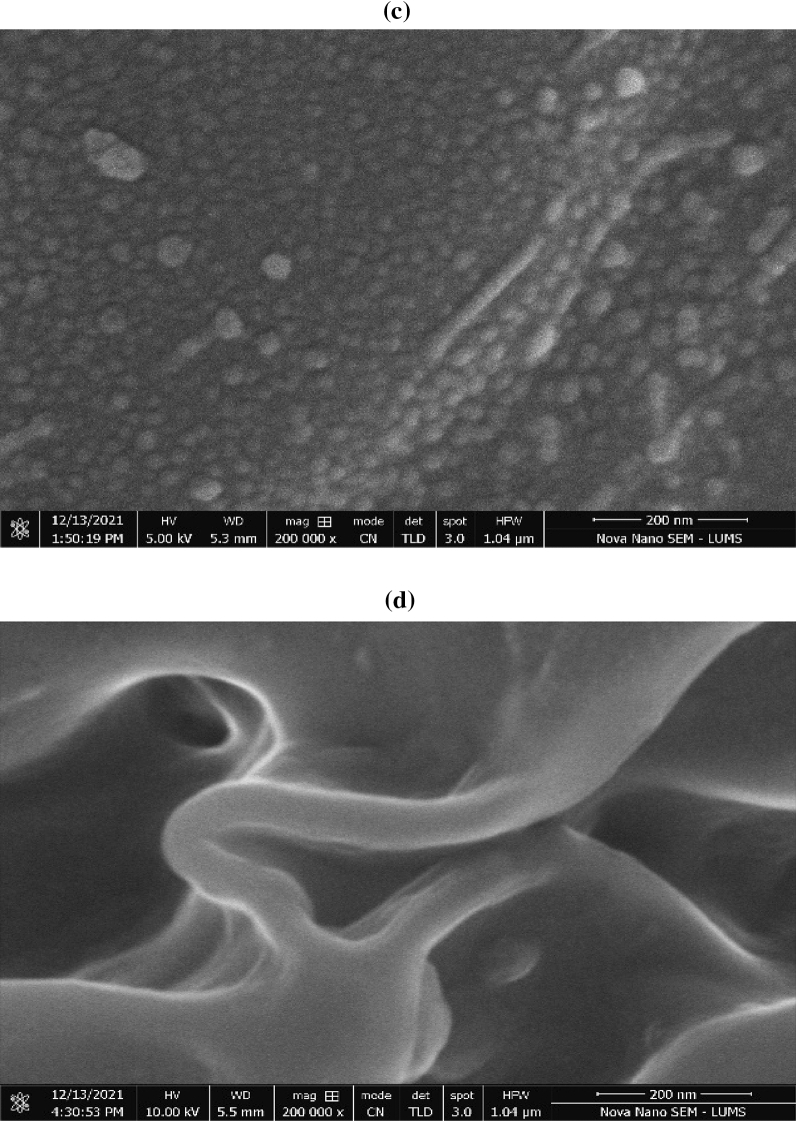
SEM images of manufactured nano fertilizers (**a**) nonimmobilized control (T2), (**b**) immobilized control (T3), (**c**) NI/Pro-MNF (T4), (**d**) I/Pro-MNF (T6).

The SEM images of control and immobilized control are shown in Fig. 6a, b, respectively. In Fig. 6a the block-like structure with distinct edges were observed in control fertilizers morphology. Figure 6a, b showed that there was no agglomeration or cluster formation because there was no complexing agent present in them. Figure 6b, demonstrated that the immobilized control's morphology has a complicated agglomeration of particles. Besides, it is evident from the images that the immobilized substance (sodium alginate) has effectively sorbed the control fertilizer onto the surface. The prior studies have demonstrated the superiority of sodium alginate as a material for the immobilization of components, which primarily occur through the sorption process^65^. Figure 6a, b obtained at nanometer scales demonstrate that nano-fertilizer was successfully synthesized as many of the particles are visible in this range.

The SEM images of NI/Pro-MNF and I/Pro-MNF are presented in Fig. 6c, d, respectively. Both SEM images of NI/Pro-MNF and I/Pro-MNF were used to find out the difference between surface and morphological characteristics. The morphology of NI/Pro-MNF (Fig. 6c) shows the spherical-like shape and some of oval shape along with soft macroscopic separations. Furthermore, the macroscopic interspaces between the particles, clearly show that the complexing agent (proline) has been well dispersed with aggregated small particles presence which are spread on the surface^66^. In Fig. 6d the morphology of the I/Pro-MNF showed that it possessed a smooth layered structure with complex aggregation. Besides, it is evident from the images that the immobilized substance (sodium alginate) has effectively absorbed the control fertilizer onto the surface^65^. Figure 6c, d obtained at nanometer scales demonstrate that nano-fertilizer was successfully synthesized as many of the particles are visible in this range.

#### XRD analysis

**Figure 7:**
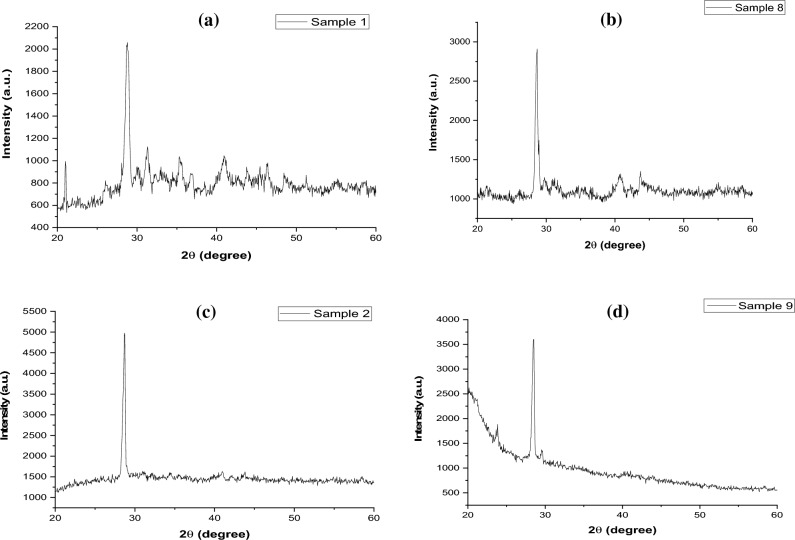
XRD spectra of (**a**) non-immobilized control (T2), (**b**) immobilized control (T3), (**c**) NI/Pro-MNF (T4), (**d**) I/Pro-MNF (T6).

When the crystalline size reduced into the dimensions of nanosized from a bulk material, broadening of XRD peaks occurred. The Scherrer equation, D = κλ/(β θ cos) is particularly used to determine quantitatively the broadening of peak at diffraction angle (θ), which is related to the width of the peak at half of its height (β) and the crystalline domain size (D)^67^. The Scherrer constant, κ, is typically considered to be 0.9^68^ but the morphology of crystal domain can change the value of Scherrer constant, κ. The wavelength (λ) is dependent on the used type of X-rays. In the Scherrer equation, the diffraction angle is in radians (not degrees) and corresponds to θ and not 2θ as is typically plotted in an XRD pattern. The crystalline domain size does not necessarily correspond to particle size, as particles can be polycrystalline, containing multiple crystalline domains^69^. The average particle size of control and immobilized control nano-fertilizer were found to be 24.49 nm and 24.50 nm, respectively^70,71^. The average particle size of NI/Pro-MNF was determined to be 27.75 nm whereas the average particle size of I/Pro-MNF was 37.81 nm^70,71^ (Fig. 7).

## Conclusions

Complexation of micronutrients with complexing agents reduce undesirable reactions of fertilizers in soil water system. These encapsulated fertilizers are applied to plants for long period of time because that polymeric material degrades biologically, and release loaded nutrients according to the plant needs. So, applying fertilizer to field once connected with significant cost reductions as well as the potential to lower labor expenses. In present work an effort was made to address all above points by synthesizing immobilized and non-immobilized complexed nano fertilizers. It was revealed through SEM and XRD analysis that the size of manufactured fertilizers was between 1 and 200 nm. In NI-Pro-MNF stretching vibration peaks at 1600.9 cm^−1^ (C=O), 3336 cm^−1^ (N–H) and at 1090.2 cm^−1^ corresponds to the pyrrolidine ring's N–H in a twisting and rocking vibrations are the evidence of complex formation of metal ions with complexing agent proline. FTIR spectrum of I/Pro-MNF show that nano complexes successfully loaded or encapsulated because Na–O bond vibration was associated with the peak that appeared at 1000 cm^−1^. Impact of these prepared fertilizers was checked on basil plants. Basil plants, in addition to serving as a garden ornament, is served as a source of essential oil (EO) used in food, fragrance, and flavor. Additionally, compared to non-immobilized nano-fertilizers, immobilized nano-fertilizers were seen to generally improve the growth parameters of basil plant. The increased EO yield from 0.0035 to 0.1226% and other plant growth parameters in basil plants after applying various synthesized nano-fertilizers demonstrate how crucial agricultural nutrient management is for the growing of basil plants. Improvement in quality and quantity of crops is possible by availability of adequate level of nutrients according to plant requirements, soil nature and peak harvesting time.

## Data Availability

All data has been included in the manuscript. If any other data required relevant to publication, corresponding author would provide that.
